# Comparative Analysis of Bacterial Communities in a Potato Field as Determined by Pyrosequencing

**DOI:** 10.1371/journal.pone.0023321

**Published:** 2011-08-19

**Authors:** Özgül İnceoğlu, Waleed Abu Al-Soud, Joana Falcão Salles, Alexander V. Semenov, Jan Dirk van Elsas

**Affiliations:** 1 Department of Microbial Ecology, Centre for Life Sciences, Nijenborgh 7, Groningen, The Netherlands; 2 Department of Biology, University of Copenhagen, Copenhagen, Denmark; Argonne National Laboratory, United States of America

## Abstract

**Background:**

Plants selectively attract particular soil microorganisms, in particular consumers of root-excreted compounds. It is unclear to what extent cultivar type and/or growth stage affect this process.

**Methodology/Principal Findings:**

DNA-based pyrosequencing was used to characterize the structure of bacterial communities in a field cropped with potato. The rhizospheres of six cultivars denoted Aveka, Aventra, Karnico, Modena, Premiere and Desiree, at three growth stages (young, flowering and senescence) were examined, in addition to corresponding bulk soils. Around 350,000 sequences were obtained (5,700 to 38,000 per sample). Across all samples, rank abundance distributions best fitted the power law model, which indicates a community composed of a few highly dominant species next to numerous rare species. Grouping of the sequences showed that members of the *Actinobacteria, Alphaproteobacteria*, next to as-yet-unclassified bacteria, dominated. Other groups that were consistently found, albeit at lower abundance, were *Beta-*, *Gamma-* and *Deltaproteobacteria* and *Acidobacteria*. Principal components analyses revealed that rhizosphere samples were significantly different from corresponding bulk soil in each growth stage. Furthermore, cultivar effects were found in the young plant stage, whereas these became insignificant in the flowering and senescence stages. Besides, an effect of time of season was observed for both rhizosphere and bulk soils. The analyzed rhizosphere samples of the potato cultivars were grouped into two groups, in accordance with the allocation of carbon to starch in their tubers, i.e. Aveka, Aventra and Karnico (high) versus Premiere and Desiree (low) and thus replicates per group were established.

**Conclusions:**

Across all potato cultivars, the young plant stages revealed cultivar-dependent bacterial community structures, which disappeared in the flowering and senescence stages. Furthermore, *Pseudomonas*, *Beta-*, *Alpha-* and *Deltaproteobacteria* flourished under different ecological conditions than the *Acidobacteria*.

## Introduction

Soil microbial communities are strongly influenced by plant roots, which mainly results from, among other factors, the excretion of organic compounds in root exudates. Via this active release of carbonaceous compounds, plants can selectively attract particular soil microorganisms to their rhizospheres, including the primary consumers of the root-excreted compounds [1]–[3]. Thus, soil microbial communities can be important determinants of plant growth and health, via the stimulation of root growth and the protection of plants against phytopathogens. Given the basic plant effect on soil, microbial communities are expected to differ in the rhizospheres of different plants or cultivars if the latter exhibit different root exudation patterns. The level and quality of root exudation may be an inherent property of a plant genotype and may also be affected by the local soil conditions as well as the stage of plant development [4]–[6]. Consequently, plant-associated bacterial communities, as well as the dynamics of their changes, can be different between cultivars of the same plant species [7]. In a recent PCR-DGGE based study on the dynamics of the bacterial communities in the potato rhizosphere, we tentatively linked the differences in the community structures to different plant physiologies [8]. However, since PCR-DGGE only allows an assessment of the dominant members of microbial communities in natural systems [9], the extent to which this effect holds true for the less abundant plant-associated bacterial species remained unknown.

The currently-available massive parallel pyrosequencing (using 16S rRNA genes) of environmental DNA allows the rapid analysis of microbial communities at a much higher throughput than has previously been possible [10]–[13]. The reads provided by pyrosequencing have been shown to yield taxonomical information with considerable resolving power [14], [15], thus allowing us to establish the relative abundances of different members of the microbial communities under study. Although, in the light of the biases that are inherent to molecular approaches to soil, the representation of pyrosequencing data can be questioned [16], the method in principle contributes to the molecular toolbox that may address whether microorganisms follow the ecological rules that have been described for macro-organisms like plants [17], [18]. On the basis of the extensive sequence data, rank distribution patterns can be modeled – using log normal, truncated log, geometric or power law distribution models - in so-called rank abundance distributions (RADs), which might provide an integrated understanding of the underlying ecological rules that govern microbial diversity and abundance [19]–[21]. In this context, recent results, which were based on large bacterial data sets, revealed that both the power law [22] and log-normal distributions [20], to a similar extent, could explain the typical hollow curves that are often found for soil bacterial communities [21].

The boost in information available due to the deep sequencing of environmental DNA has also allowed microbial ecologists to describe microbial systems in terms of shallow- as well as deeply-branching bacterial lineages (from phyla to genera or even species ). On the basis of the data, one may then interrogate whether these lineages are ecologically coherent, i.e. whether they share similar life strategies or traits that tell them apart from other, more distantly-related, taxa. Similarly, it has been suggested that some taxa are ecologically very coherent. For instance, such taxa may be broadly classified as having either r-type (*Betaproteobacteria* class and *Pseudomonadales* order) or K-type (phylum *Acidobacteria*) life strategies [23], [24].

In the current study, we assessed the dynamics of the structures of the bacterial communities that inhabit the rhizospheres of different potato cultivars during growth in an experimental field in comparison with those in corresponding bulk soil. The sequence information obtained allowed us to build hypotheses with respect to the effect of the rhizospheres of the different cultivars, over time, on the associated bacterial communities. Besides, we performed tests of the distributions of taxa at the genus level, using five different distribution models.

## Results

### Plant growth across the field

The growth of the different potato cultivars in the field over the 2008 growth season has been described previously [8]. As indicated in Materials and Methods, all cultivars, in terms of their growth in the field, fell into two groups, i.e. the early (Premiere - P, Desiree - D) versus late (Aveka - A, Aventra - Av, Karnico - K, Modena - M) cultivars. This coincided with the allocation of starch to their tubers. It was also observed that cultivars P and D had shorter roots (around 15 cm) whereas all other cultivars had longer root systems, allowing deeper rooting (average root length around 25 cm). No signs of disease or nutrient limitation were observed for any of the potato cultivars over the entire growth season (2008) in all plots.

### Pyrosequencing data – statistical analyses

We obtained a total of 359,694 sequences from the 20 samples that were analyzed. Over 99% of the sequences were bacterial (see Material & Methods), and all non-bacterial sequences were removed from the analyses. The read numbers were uneven, ranging from 5,736 to 38,000 per sample. Following filtering and removal of noise (See M&M), 313,258 sequences (4,500 to 35,000 per sample) remained. These were used for further analyses.

**Figure 1:**
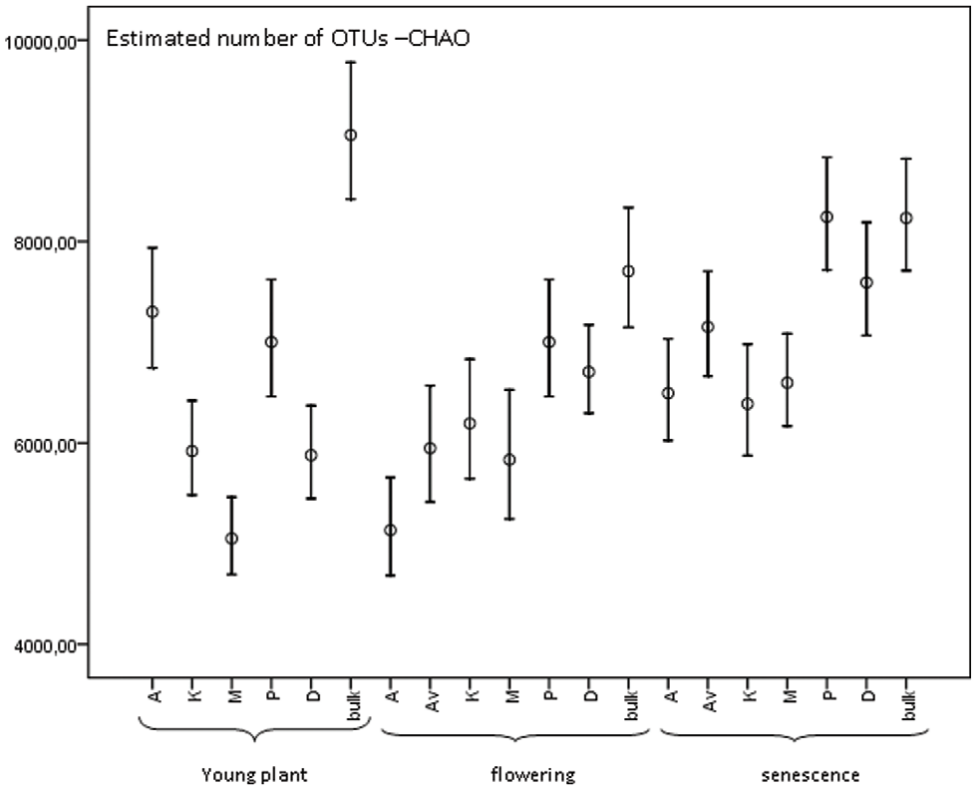
CHAO1 estimations over the season. 4,000 to 6,000 sequences per sample were randomly taken to compare samples in the same range for CHAO1 analysis (harmonized data). 95% confidence intervals are shown. (A) Aveka, (Av) Aventra, (K) Karnico, (M) Modena, (P) Premiere, (D) Désirée.

However, since estimated OTU numbers increased with increasing number of sequences, these analyses, including rarefaction, were primarily performed on the basis of a “harmonized” data set, on several occasions compared to the full data set (see Material & Methods). The rarefaction analysis revealed that plateau levels (indicating complete sampling) were reached in none of the samples. Furthermore, 55,121 OTUs (defined using a 97% cut off value) were found, on the basis of all data, in the complete data set. The CHAO1 estimator of OTU richness in this case predicted richness values in the range 5,134 to 8,730 per 1-g sample (Fig. 1), exceeding the estimation of 4,000 OTUs in 1 g of soil made by Torsvik et al. [25].

Based on the CHAO1 richness estimator, the richness in the bulk soil samples revealed a slightly decreasing trend from the young (9,058) to the senescence stage (8,234). Thus, the highest richness values were found in the bulk soils in the young and flowering stages (Fig. 1). Also, in the young plant stage, the bulk soil sample showed higher richness (9,058) than any of the rhizosphere samples (max. 7,301), indicating that the potato rhizospheres at this stage exerted selective pressure on the soil bacteria resulting in a reduced niche breadth (Fig. 1). This difference in richness between bulk and rhizosphere samples decreased over the season. In fact, in the senescence stage no difference in richness was observed for the rhizosphere in comparison to bulk soil samples. At none of the sampling times was any cultivar effect observed.

### Rank abundance distributions (RADs)

RADs were plotted for the harmonized data sets (as well as for all data, after removal of unclassified ones), to assess the relative abundances of established genera and to ascertain whether there were differences between the curves describing the RADs. The numbers of identifiable genera ranged between 112 and 3,004 across the samples (whereas this range was 1,246 to 13,802 for the total data set).

**Figure 2:**
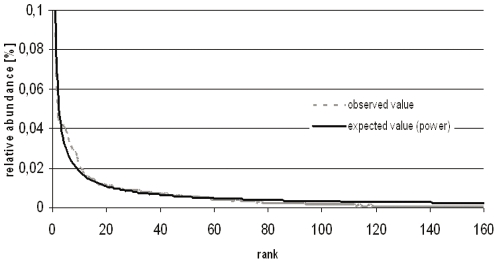
Rank abundance distribution (RAD) for rhizosphere of Aveka in the flowering stage. Power law fitted best.

Fitting of the observed distributions (based on relative abundance) to mathematical models indicated that none of our RADs could be fitted by either the broken stick or the geometric model. In contrast, fifteen out of the twenty distributions could be fitted by the log normal model, and fourteen by the truncated log model (Table S2). Strikingly, fitting of the RADs to the power law model resulted in significant fits (p<0.01) of all, with average pseudo-R^2^ values of 0.98±0.01 (Fig. 2).

The values of the shape parameter *m* were calculated and compared for the harmonized data set (Table 1). There was a significant effect of rhizosphere on *m*, these values varied between −0.77 and −0.64 (higher *m* values indicate a higher evenness of the distribution). In the young plant stage, all samples revealed statistically similar (P>0.05) *m* values, ranging from −0.77 (P) to −0.69 (M). At flowering stage, three different statistical classes of *m* values were discerned, i.e. for cultivar A ( lowest *m*, i.e. −0.74±0.01), P, D, K and M (*m* around −0.070) and Av (highest *m*, i.e. −0.62±0.04). Finally, the senescence stage was characterized by overall similar *m* values around −0.70. A comparison per sample over time revealed that in four of the six samples the *m* value remained statistically similar across time (P>0.05), whereas in two samples, cultivars Av and D, a significant shift towards a lower evenness at senescence stage was noted (Table 1).

**Table 1:** Parameters of the power law distribution calculated across all samples *m* values indicate evenness of the distribution.

samples	young plant	flowering	senescence
Aveka	−0,70^a^ ^A^±0.07	−0,74^a^ ^A^±0.01	−0,70^a^ ^A^±0.01
Aventra	ND	−0,62^a^ ^B^±0.04	−0,70^b^ ^A^±0.02
Karnico	−0,73^a^ ^A^±0.07	−0,70^a^ ^C^±0.02	−0,70^a^ ^A^±0.01
Modena	−0,69^a^ ^A^±0.02	−0,70^a^ ^C^±0.02	−0,71^a^ ^A^±0.03
Premiere	−0,77^a^ ^A^±0.07	−0,71^a^ ^C^±0.02	−0,72^a^ ^A^±0.01
Desiree	−0,70^a^ ^A^±0.02	−0,69^a^ ^C^±0.03	−0,73^b^ ^A^±0.02
Bulk	−0,69^a^ ^A^±0.04	−0,74^a^ ^A^±0.06	−0,70^a^ ^A^±0.03

Standard errors of the mean are indicated. Statistical classes were determined across cultivars/bulk soil per time point as well as across time per cultivar/bulk soil. Different letters represent statistically different subgroups:

a,b significant (P<0,05) differences across growth stages per cultivar or per bulk soil.

A,B significant (P<0,05) differences across cultivar/bulk soil per growth stage.

### Bacterial community dynamics

**Figure 3:**
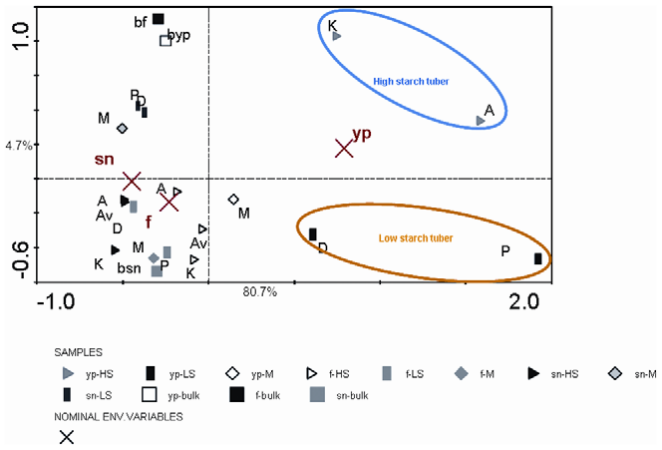
Ordination biplots generated by principal component analysis (PCA) of bacterial communities based on the genus level, in the rhizosphere of potato cultivars with different starch content and corresponding bulk soil, at different growth stages. Centroids indicate the effect of growth stage on the community structure. The eigenvalues displayed on the diagram axes refer to the percentage variation of the respective axis. (yp) young plant, (f) flowering, (sn) senescence, (A) Aveka, (Av) Aventra, (K) Karnico, (M) Modena, (P) Premiere, (D) Désirée, (b) bulk, (HS) high starch tuber, (LS) low starch tuber.

In order to examine the effect of cultivar and plant growth phase on the total distribution of phyla and genera, we performed a PCA on all data, using CANOCO (Microcomputer Power, Ithaca, NY). When plant growth stage was examined as an explanatory variable, the young stage revealed the highest degree of variation of the bacterial community structures between cultivars, while the flowering stage had a lesser effect (lower vector magnitude), being more closely related with the bacterial communities at senescence stage (Fig. 3). A clear effect of plant growth was observed, as the rhizosphere samples from the young plant stage were quite different from those at flowering and senescence stages (Fig. 3, Fig. S1). In Figure 3, an effect of cultivar type (high- versus low-starch tuber) can also be observed in the young plant stage along the second axis, whereas this effect was reduced in the subsequent plant growth stages.

### Bacterial community composition

**Figure 4:**
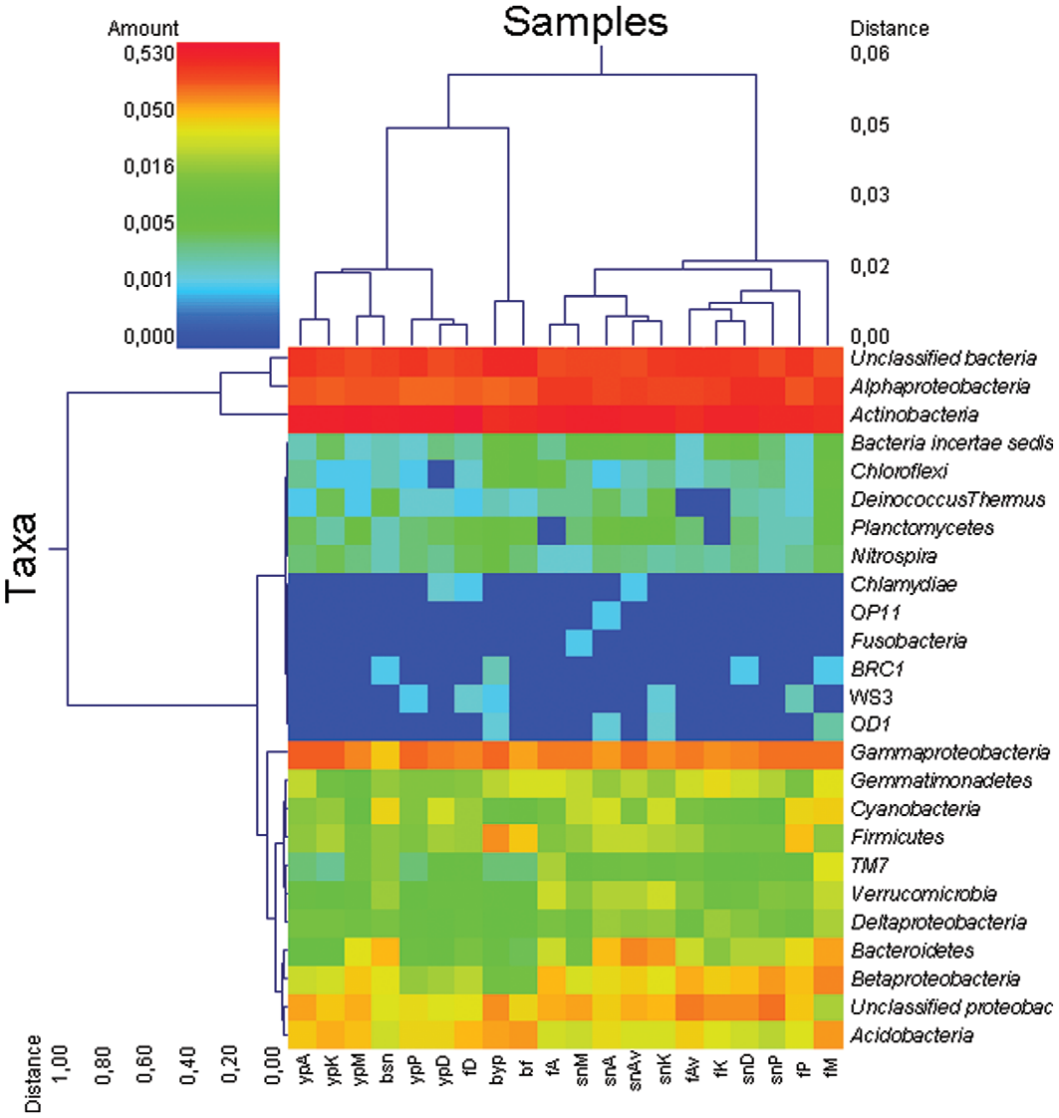
Double dendrogram, based on the Ward minimum variance clustering method for major phyla and class investigated using 16S rRNA gene pyrosequencing. The heat map indicates the relative percentage of each phylum/class within each sample. On top of the figure, the distance of the samples based on weighted pair linkage and Manhattan distance methods with no scaling is shown, along with a distance score. (A) Aveka, (Av) Aventra, (K) Karnico, (M) Modena, (P) Premiere, (D) Désirée, (b) bulk, (yp) young plant, (f) flowering, (sn) senescence.

Overall, 25 phyla were found across the samples. Strikingly, 15–35% of the sequences remained unclassified, as these were below the 80% threshold, indicating that they belong to as-yet-uncultured/unrecognized bacteria. Subsamples consisting of 100 sequences were taken from the unclassified sequences of three bulk and three rhizosphere soil samples. Per subsample (soil or rhizosphere), trees were built and the clustering was analyzed. In all cases, most (>95%) of the sequences fell in 7–10 branches, in which individual reads often showed deep branching. “Flat” branches containing more than 5 sequences were never observed using the 97% cut-off level, indicating that none of the tested sequences showed overall dominance (i.e. roughly >1.3% of the total). The relative abundances of specific bacterial groups were studied at different taxonomic levels, i.e. phylum, class, order and genus. The analyses revealed that *Actinobacteria* and *Alphaproteobacteria* were the most abundant groups (8–50% of total sequences), followed by *Gammaproteobacteria*, *Betaproteobacteria*, *Acidobacteria*, *Gemmatimonadetes*, *Firmicutes*, *Verrucomicrobia*, *Deltaproteobacteria*, *Cyanobacteria*, *Bacteriodetes* and the TM7 group (1–5%), and the least dominant phyla (<0.1%) *BRC1, Fusobacteria* and others (Fig. 4).

We then used the grouping of cultivars based on plant physiology and tuber starch content (A, Av and K - high starch tuber; P and D - low starch tubers, M – modified high starch tuber) to assess the effects of plant physiology on the abundance of different plant-associated bacteria (an analysis per cultivar can be found in Text S1, Fig. S2).

**Figure 5:**
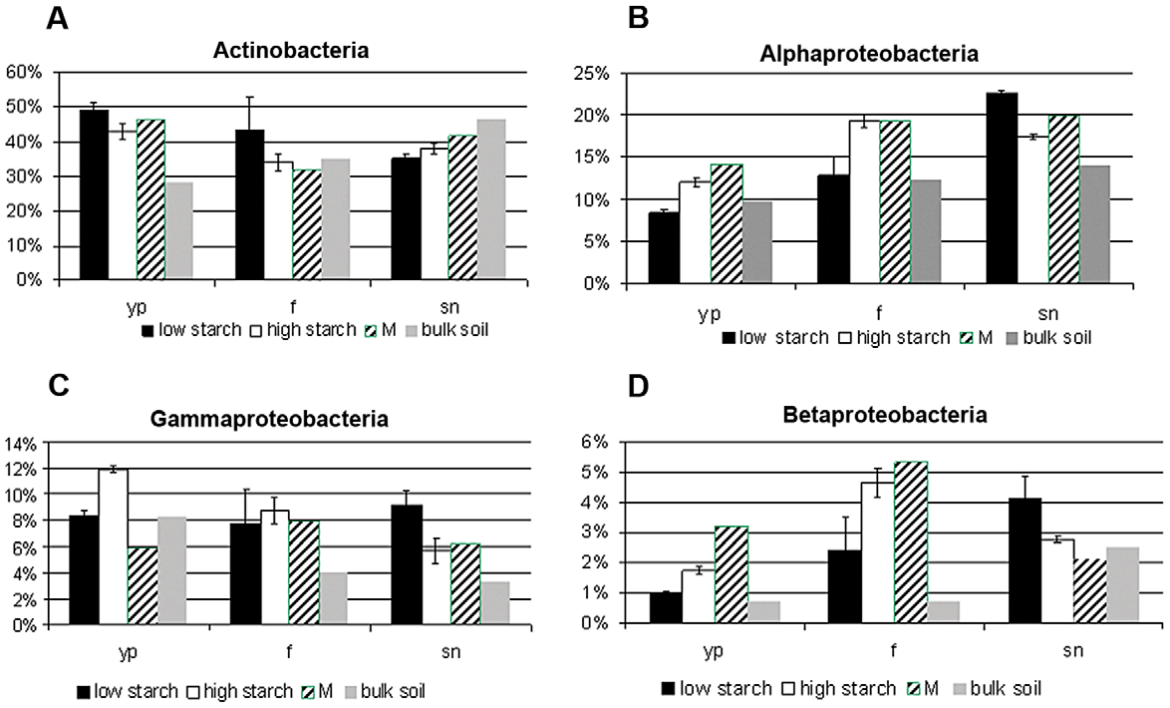
Relative abundance of (A) Actinobacteria, (B) Alphaproteobacteria, (C) Gammaproteobacteria and (D) Betaproteobacteria in the rhizosphere of potato cultivars with different starch content and corresponding bulk soil, at different growth stages. Error bars indicate standard errors. (yp) young plant, (f) flowering, (sn) senescence. Black bars – low starch tuber, white bars – high starch tuber, dashed bars – (transgenic) cultivar M, grey bars – bulk soil.

The most abundant bacterial groups showed - in general - increases in relative abundance in the rhizospheres of different cultivars in comparison to bulk soil, although the differences were not large. Yet they were consistently observed across the growth season. First, in the young growth stage, the relative abundance of *Actinobacteria* was higher in the rhizospheres of both low-starch-tuber (49%) and high-starch-tuber (42%) potatoes than in corresponding bulk soil (28%). However, this effect disappeared in the flowering and senescence stages (Fig. 5A). In addition, the high-starch-tuber cultivars showed positive rhizosphere effects for *Alphaproteobacteria* in all growth stages, whereas the low-starch-tuber cultivars only showed this effect in the senescence stage (Fig. 5B). Similarly, the high-starch-tuber cultivars revealed, in all growth stages, raised relative abundances of *Gammaproteobacteria*, whereas in the low-starch-tuber cultivars, such effects were only seen in the flowering and senescence stages (Fig. 5C). Concerning the *Betaproteobacteria*, the high- and low-starch-tuber cultivars also showed rhizosphere effects, in particular in the flowering stage (Fig. 5D). Cultivar M affected the community structures of the *Alpha*-, *Gamma*- and *Betaproteobacteria* only in the young plant stage differently in comparison to the other cultivars and the bulk soil. Interestingly, this cultivar showed the highest relative abundances of *Alpha-* and *Betaproteobacteria* (P<0.05; Fig. 5B, D) and the lowest relative abundance of *Gammaproteobacteria* overall in the young plant stage (Fig. 5C).

The relative abundance of the phylum *Acidobacterium* did not show any significant difference between rhizosphere and bulk soil in any growth stage. However, there was a clear effect of sampling time both on the rhizosphere and bulk soil values. In fact, the acidobacterial relative abundance decreased from flowering to senescence stage both in the rhizosphere and bulk soil samples (Fig. 4).

**Figure 6:**
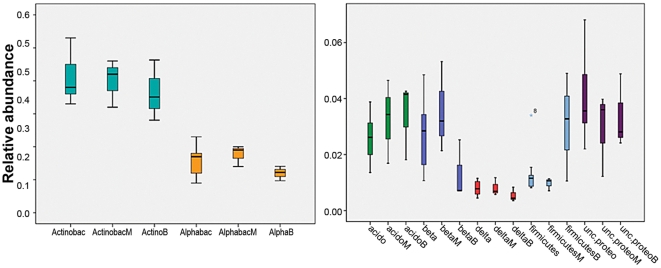
Normal operating range (NOR) of the bulk and rhizosphere soil against the genetically modified plant (M) across the season (box whisker plots). Boxes show the upper (75%) and the lower (25%) percentiles of the data. Whiskers indicate the highest and the lowest values (SPSS statistics 16). M: Modena, B: bulk, Actino: Actinobacteria, Alpha: Alphaproteobacteria, Acido: Acidobacteria, beta: Betaproteobacteria, delta: Deltaproteobacteria, unc. proteo: uncultured Proteobacteria.

A so-called normal operating range (NOR) was concocted to visualize the fluctuations in the rhizosphere samples over the season and to determine whether those at the transgenic cultivar M differed from those at the other (unmodified) potato cultivars. The borders of the NOR were established by the maxima and minima, i.e. the upper (75%) and the lower (25%) percentiles of the relative abundances of phyla and or class, the average of five cultivars cultivar M and bulk soil, including all sampling times. We thus assessed whether the fluctuations in these values at cultivar M fitted within the NOR (in relation to the rhizosphere or the bulk soil) and found that the maximal relative abundances of the *Acidobacteria* (and possibly *Betaproteobacteria*) exceeded the NOR whereas *Firmicutes* and uncultured *Proteobacteria* remained below the set NOR (Fig. 6). Other groups fitted within the borders of the NOR. In figure 5, it is also seen that the *Firmicutes* in the bulk soil were strikingly higher than in the rhizosphere, indicating a negative rhizosphere effect.

### Classification of genera

Overall, 450 genera were found across the samples. The ten most abundant genera from each of the 20 samples were then classed as a percentage of the total sequences per sample (Table S3). This analysis showed that there were 33 dominant genera in total, including eight members of the *Actinobacteria*, six of the *Acidobacteria*, five *Alphaproteobacteria*, four *Gammaproteobacteria*, three *Bacteriodetes*, two *Chloroflex*i and one each of the *Cyanobacteria*, *Verrucomicrobia*, *Firmicutes* and *Gemmatimonadetes*. In particular, Rhodanobacter was observed in all bulk as well as rhizosphere soil samples from the young (8–26%) and flowering stages as one of the ten abundant genera. However, this genus was not detected in the rhizosphere at senescence stage. *Solibacterales* (*Acidobacteria*, *Group 3*) were found in all samples as one of the ten most abundant genera, except the rhizosphere of cultivar K at flowering stage. Although present in most rhizosphere samples, the relative abundance of *Solibacterales as* well as of *Acidobacterium Group 1* types were always higher in the bulk soil, at the young and flowering stages, than in rhizosphere samples. In addition, the relative abundance of Solibacterales in the rhizosphere at senescence stage was roughly similar to that in the corresponding bulk soil. *Gemmatimonas*, *Acidobacterium Group 1* and 3 and *Serratia* constituted the most dominant genera in the bulk soil collected during the young plant and flowering stages, whereas *Streptophyta*, and *TM7 incertae sedis* were the most dominant genera in the senescence stage (Fig. S3).

In order to analyze whether the distribution of the low-abundance genera differed between samples, sequences with abundances below 0.1% were analyzed. A total of 425 of such low-abundance genera were found. In the young plant stage, only 292 of these were observed, leaving 133 undetected. Of the 133 types not found at the young stage, 90 were exclusively observed at flowering stage, 18 only at senescence stage (both in rhizospheres), whereas 17 genera were unique for bulk soil. Twenty-nine % of the genera appeared only once and only 2% of the genera appeared in more than 50% of the samples.

### Ecological indices

**Figure 7:**
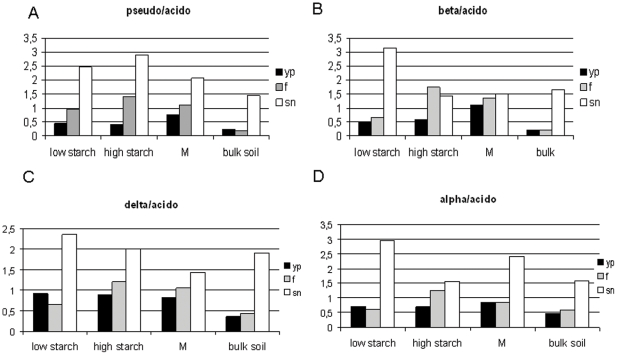
Ratio (F) of relative abundances of different groups versus Acidobacteria (A) Pseudomonas/Acidobacteria, (B) Betaproteobacteria/Acidobacteria, (C) Deltaproteobacteria/Acidobacteria, and (D) Alphaproteobacteria/Acidobacteria.

Some phyla and/or classes are thought to exhibit either mainly r-type/copiotrophic (*Betaproteobacteria*, *Pseudomonas*) or K-type/oligotrophic (*Acidobacteria*) ecological behaviour. Consequently, such groups may reveal higher abundances in soils with higher versus lower levels of easily-available carbon, respectively [23], [24]. In the current study, we found that *Pseudomonas* types apparently preferred different plant growth stages than *Acidobacteria*, since the ratio's between these groups (F_pseudo/acido_) were growth stage-dependent and increased from the young plant to senescence stage, both in rhizosphere and bulk soils. Specifically, the abundances of the *Acidobacteria* were mainly constant, whereas those of *Pseudomonas* spp. changed over the season. For instance, F_pseudo/acido_ was around 6 times higher in bulk soil collected at the senescence stage than in bulk soils at the young and flowering stages. Since both *Pseudomonas* and *Betaproteobacteria* have been suggested to be largely copiotrophic, we expected to see a similar pattern for F_beta/acido_. Whereas this was partially true, a strong shift of F_beta/acido_ in the senescence stage of high-starch-tuber cultivars (and cultivar M) was not observed, as in F_pseudo/acido_ (Fig. 7A, B). Finally, the *Alpha-* and *Deltaproteobacteria* revealed divergent dynamics compared to the *Acidobacteria*, similar to *Pseudomonas* (Fig. 7C, D).

## Discussion

Soil microbial communities can be affected by soil characteristics, environmental conditions and crop management strategies like crop rotation and residue removal [26]–[29]. Rhizosphere microbial communities are also known to be governed by the complex interactions driven by soil type, plant species (genotypes) and growth [30]–[32]. In this study, we assessed - by direct pyrosequencing of environmental DNA - the dynamics of the relative abundances in soil and the community structures of soil bacterial communities as a function of cultivar type and growth stage, using six potato cultivars in one experimental field. It was hypothesized that, per cultivar and growth stage, potato roots may select for different bacterial groups in the rhizosphere.

Indeed, a striking observation of this study was that the young plant stage stands out as being a unique determinator of community structure in the rhizosphere, as compared to the other two plant growth stages. This was shown using the RADs, community analysis and also the ecological indices. First of all, let us consider the RADs for all cultivars and vegetation stages. The power law model, which is characteristic for complex systems, where multiple processes play important roles in shaping species abundance distributions [21], [33], was found to fit all RADs. In other studies, this model also proved to be quite suitable to describe RADs for plants in soil [20], [22], [34]. This may indicate that the distribution of plants and belowground organisms is similarly structured, in contrast to distributions of e.g. animals [35]. Across the different bulk and rhizosphere soils, the senescence stage had the most stable evenness across all cultivars and bulk soil. Besides, our PCA analyses showed that the most divergent community structures occurred in the young plant stage, whereas the flowering and senescence stages revealed increasingly similar community structures. The young plant stage is apparently a cultivar-specific stage, given the fact that a dichotomy between cultivars was observed. This effect became less dominant in the following stages.

Due to technical (e.g. low sequence numbers) and logistic constraints, studies on soil microbial diversity and functioning often mainly focus on the most dominant species. However, some rare species may have large effects on soil functioning, in spite of their low total biomass or rareness [36], [37]. Here, pyrosequencing provided the unique opportunity to access the less abundant bacterial taxa under potato, as well as to compare their presence across the cultivars at different growth stages. Interestingly, some functionally important yet less abundant genera found, such as *Burkholderia* and *Nitrospira*, were clearly growth-stage-specific. This might be an indication of a plant growth effect due to the different exudation pattern as a result of differing root types in the early developmental stages. High-starch-tuber cultivars had longer roots than low-starch-tuber ones. The high variability in bacterial community structure at young plants was also previously demonstrated, when comparisons of community structure and function were undertaken at specific time points for different plant species [38], [39].

Moreover, dominant members of particular phyla or classes were used as ecological indicators to reveal prevailing ecological behavior (e.g. along the copiotroph/oligotroph scale). Although it is rather implausible that a complete phylum would have shared ecological characteristics, our indicators revealed a dynamics (using the ratio's F_beta/acido_, F_alpha/acido_, F_delta/acido_ and F_pseudo/acido_) indicative for interpretable responses of the *Beta-*, *Alpha-*, *Deltaproteobacteria* and *Pseudomonas* spp. versus the *Acidobacteria* to shifting ecological conditions in the soil. This was thought to reflect the plant growth stages and conditions in the corresponding bulk soils. All these ratios were lower in the young plant stage than in the other stages. In this context, it should be stated that the general prevalence of many members of the *Acidobacteria* in bulk soils (as opposed to rhizosphere soils) may relate to their generally oligotrophic (K strategist) lifestyle [23], [24]. In contrast, many of the *Betaproteobacteria* [23] and *Pseudomonas* species [24] are known as typical copiotrophs (r strategists) which tend to be strongly favored under nutrient-rich conditions. The relationship between the *Betaproteobacteria* or *Pseudomonas* and the *Acidobacteria* on the other hand thus yields ecologically-meaningful parameters, which allow to give interpretational value to the data [23]. In practical terms, each plant growth stage may be characterized by a specific but different root exudation pattern which drives different bacterial communities. A plausible explanation for such an effect may come from the observation of the general increase in the ratios in the rhizosphere during the growth season. During the flowering and senescence stages, the rhizosphere may have offered extra nutrient-rich niches, whereas more nutrient-deprived conditions may have occurred in the young plant stages. In bulk soil, the early (young) stage was preferred by oligotrophs (Fig. 5) whereas the late (senescence) stage was abundant with copiotrophs. An explanation might be that in the senescence stage, bulk soil may have experienced more pronounced nutrient suppletion than at earlier stages: both below- and aboveground plant material may have started to enrich the surrounding soil environment with nutrients. In our observations, *Pseudomonas* was found independently of cultivar, whereas the class *Betaproteobacteria* was likely cultivar-dependent. It is here suggested that the shift from oligotrophs to copiotrophs was least pronounced in cultivar M. In fact, the bacterial community composition in cultivar M revealed a more stable equilibrium state between oligotrophs and copiotrophs during the season than in the other cultivars. Besides the former two taxa, *Alpha-* and *Deltaproteobacteria* have also been suggested as being grossly copiotrophic; this was supported by their behavior being largely similar to *Pseudomonas* over *Acidobacteria*.

In the current pyrosequencing-based analysis of bulk and potato rhizosphere soil, not a single sample was dominated by members of the *Acidobacteria*, which ranged from a mere 1.3 to 5% of the total. One of the striking discoveries in surveys of soils has been the rather frequent detection, and dominance, of sequences of *Acidobacteria* [40]. However, not much is known about the physiological capabilities and habitat preferences of the underlying organisms [41], [42]. Pyrosequencing of different soils has shown a variation in the relative abundance of *Acidobacteria* from 2.4% to an overwhelming 78.5% [13]. Moreover, a strong correlation between the relative abundance of soil *Acidobacteria* and soil pH was found. Soils with pH around 5.5 were indicated to contain around 10% *Acidobacteria*, which contrasts with our study in the pH-5.5 Buinen soil, where this value was 4%. This difference might be due to soil type and management, since agricultural soil was used in our study, whereas forest and grassland soils were used in the previous study [13]. Using a new q-PCR system for detection of the acidobacterial *Holophaga* and/or *Luteolibacter/Prosthecobacter* spp., Nunes da Rocha et al. recently showed that some of these bacteria do exhibit a preference for the rhizosphere (in this case, of leek) [43]. However, there are other studies which indicate that the relatively high carbon availability in the rhizosphere supports (other) fast-growing microorganisms, which potentially outcompete members of the *Acidobacteria* [44]. In our community analyses, *Acidobacteria* had higher relative abundances in the bulk than in the rhizosphere soils (Table S3). The discrepancy between the two studies might be the result of the type of plant studied and/or the use of a different detection system. The leek rhizosphere might represent a more nutrient-limited or even a less competitive environment in contrast to the potato rhizosphere. Besides, group-specific primers may lead to different results in comparison to general bacterial primer systems.

We previously reported on bacterial community changes in the same potato field, on the basis of PCR-DGGE and clone libraries [8]. PCR-DGGE can detect bacterial populations in soil that make up roughly 0.1–1% or more of the total community that is present [9]. Using pyrosequencing, we now generated tens of thousands of sequences per sample (instead of up to 100 DGGE bands or clones), which in principle provides a broader view of the bacterial communities in the samples. Overall, the pyrosequencing data confirmed our previous general bacterial PCR-DGGE based findings. For instance, we revealed community changes over time in the bulk soil, not only with respect to the overall bacterial diversity but also to the prevalence of different bacterial groups. Thus, increasing abundances of *Actinobacteria*, *Alpha-*, *Beta-* and *Deltaproteobacteria*, versus decreasing ones of *Gammaproteobacteria*, *Acidobacteria* etc, were found. On the other hand, differences that are attributable to the use of different molecular approaches were also noted.

Overall, the direct pyrosequencing used here followed the strategy recently established in previous investigations [45]. While any PCR primer set for amplification of 16S rRNA genes may miss a considerable amount of the extant microbial diversity [46], at present, no clear answer can be given with respect to the real extant community make-up. Since PCR biases particularly affect the detection of rare sequences in a sample, it is important that our data on the rare biosphere are viewed with proper caution. However, DNA amplification from complex biological samples such as soil is difficult due to the presence of PCR inhibitors, which requires optimization of PCR conditions.For instance, the use of long primers with tags and adapters (∼60 bases) directly on the original DNA can hamper the PCR and generate many aspecific bands (like we found, not shown) and even failure of DNA amplification. Based on these arguments , a previously published protocol that adds tags and adapters in a separate PCR (for 15 cycles) [47] was chosen as the most optimal one for pyrosequencing of complex environmental samples.

Our results also clearly demonstrated that a large fraction of the soil bacterial communities studied, and hence their metabolisms, still abide in an unknown territory. The “bin” unclassified bacteria was found to contain a suite of diverse taxa. As this bin dominated the community in bulk soil collected during young plant and flowering stages, this part of the soil remains a challenging reservoir of bacterial diversity. In order to understand the ecological impact of unclassified sequences, they must be identified and ideally an organism should be cultured, which once again stresses that culture-independent techniques should go hand-in-hand with culture-dependent ones. Yet, this study has provided a baseline for further characterization of changes in the bacterial community composition as determined by the potato rhizosphere, revealing direct responses of the communities to different potato cultivars and environmental conditions.

## Materials and Methods

### Field setup and sampling

An experimental field - Buinen ((B), 52°55′N-6°49E), The Netherlands, in which a completely randomized block design was set out in 2008, was used. It contained loamy sand with 5% organic matter [pH 5.0]. The field was under agricultural rotation and in the previous season, barley had been grown in it. Bulk and rhizosphere soil sampling was done as described previously [8]. For each potato cultivar, four replicate plots which were randomly distributed over the field were used. At the start of the growth season, the plots were cropped with twenty plants (tubers) each. Four plants per plot were carefully collected at each sampling. The plants with adhering soil were immediately taken to the laboratory. In the laboratory, the loosely-adhering soil on the roots was shaken off and discarded, after which the resulting roots containing rhizosphere soil were pooled per plot. Using the pooled sample, soil tightly adhering to the roots was brushed off and collected (constituting rhizosphere soil). Besides, six composite bulk soil samples, each consisting of four approximately 20 cm deep cores of each cultivar area (at least 1 m outside of the plant rows), were collected and pooled. Prior to the PCR, six bulk soil samples were pooled per growth stage. Potato cultivars Aveka [A], Aventra [Av], Karnico [K] and Modena ([M], transgenic cultivar, modified from Karnico for low amylose content) are characterized by their relatively slow growth rate and production of tubers with high starch content, whereas cultivars Premiere [P] and Désirée [D] developed faster and had tubers with relatively low starch contents [48]. For all cultivars, the young plant stage (EC30) occurred around 30 days post-planting (dpp), i.e. the end of June. However, cultivars A, Av, M and K on the one hand, and P and D on the other hand, showed different subsequent growth rates. The flowering stages (EC60) occurred between 50 and 60 dpp for cultivars D and P and between 80 and 85 dpp for cultivars A, Av, K and M (July). Finally, the senescence stages (EC99) were between 110 and 115 dpp for P and D, between 135 and 140 dpp for A and between 145 and 150 dpp for Av, K and M. All cultivars were first treated individually; in the light of the similar physiological characteristics between cultivars A, Av, K on the one hand and P and D on the other, two groups were created and further analyzed.

### Soil DNA extraction, PCR amplification and pyrosequencing

DNA was obtained from all bulk and rhizosphere soil samples using 0.5 g of homogenized soil per sample as described previously [8]. DNA qualities (average molecular sizes and purity) and quantities were estimated from gel, using the degree of DNA shearing (average molecular size) as well as the amounts of visible co-extracted compounds (quality) and a comparison to known DNA amounts (quantity) in the marker.

For the pyrosequencing, we used DNA from the individual samples. We followed an established protocol, which used an initial 30 cycle PCR [47], PCR amplification was performed using 1 U Kapa 2G hot start robust polymerase (Kapa Biosystems, US), 1× Kapa 2G Buffer, 0,3 µM of each modified primer mprk341f and mprk806r [45] and 0.3 µM dNTP mixture, 5–10 ng DNA sample in a 25 µl reaction. The mixtures were placed in a GeneAmp® PCR system 9700 cycler (Applied Biosystems, Foster, CA, USA) and thermal cycling was performed as follows: initial denaturation consisting of 2 min at 95°C; followed by 30 cycles consisting of 20 sec at 98°C, 15 sec at 59°C and 30 sec at 72°C; and final extension for 5 min at 72°C. After PCR amplification, the reaction mixes were kept on ice to prevent hybridization between PCR products and short aspecific amplicons. Analysis of PCR products on 1% agarose gel revealed bands of the corresponding size. These bands were cut using agarose gel electrophoresis and purified by a gel extraction kit (Qiagen, Netherlands). Then, the material of the individual amplifications was pooled per cultivar rhizosphere or bulk soil sample per time. On the basis of this material, a second round of PCR was performed using 1× Phusion HF buffer, 0.2 mM dNTP mixture, 0.8 U Phusion Hot Start DNA Polymerase (Finnzymes Oy, Espoo, Finland), 0.5 µM of the primers with adapters and tags (Table S1). The PCR incubation conditions were: 98°C for 30 s, followed by 15 cycles of 98°C for 5 s, 56°C for 20 s and 72°C for 20 s and a final extension of 72°C for 5 minutes. After PCR analysis on agarose gels (1%), the specific bands were cut and purified by the Montage Gel extraction kit (Millipore, Netherlands). The amplified fragments with adapters and Tags were quantified using Qubit™ fluorometer (Invitrogen, Breda, Netherlands) and mixed in approximately equal concentrations (5×10^7^ copies per µl) to ensure equal representation of each sample. These samples were subjected to a pyrosequencing reaction on one of two regions of 70_75 GS PicoTiterPlate (PTP) by using a GS FLX pyrosequencing system according to the manufacturer's instructions (Roche).

### Analysis of the sequence data

All sequences generated in this study can be downloaded from NCBI Short Read Archive, accession number: SRA036586. The sequences were aligned and filtered by the programme Mothur (http://www.mothur.org/wiki/Pre.cluster) [49]. After the filtering, we performed pre-cluster commands. This approach is based on a pseudo-single-linkage algorithm, which helps to remove sequences that are most probably due to pyrosequencing errors.

The filtered sequences were subjected to the RDP pyrosequencing pipeline in order to obtain taxonomical hierarchy [50]. Default settings of the RDP pipeline were used, with a minimum length of 150 bp [14]. The naïve Bayesian rRNA gene classifier automatically estimates the classification reliability using bootstrapping. Overall, bacterial sequences covered 99% of all analyzed sequences, and this was consistent throughout all samples. A subsample of 250 sequences which could not be assigned with bootstrap confidence was checked and these were all assigned as as-yet-uncultured bacteria of uncertain affiliation. Moreover, random subsamples of 100 unclassified sequences for three bulk and three rhizosphere soil samples from all growth stages were checked and used to construct phylogenetic trees. The evolutionary distances were computed using the Kimura 2-parameter method [51]. Rarefaction curves were also obtained for all samples by RDP pyrosequencing pipeline [50]. Since estimated sequence numbers increased with increasing number of sequences, 4,000 to 6,000 sequences per sample were randomly taken to compare samples in the same range for rarefaction and CHAO1 analysis (harmonized data). The 95% confidence intervals were also indicated by the RDP pyrosequencing pipeline.

### Statistical analyses

Principal components analysis (PCA) was applied to the relative abundance values obtained from the entire data set and harmonized data (random selection of sequences between 4000 to 6000 in triplicate) to assess whether any effect of potato cultivar and/or time (related to growth stage) could be discerned. Tests versus the harmonized data set revealed the data to be similar to those obtained with the harmonized data. CANOCO (Microcomputer Power, Ithaca, NY) was used for this analysis; using sequences grouped both at phylum and genus level. Growth stages were introduced as environmental variable. To model the distribution of sequence types (at the genus level), the log-normal, truncated-log, broken stick and geometric series models were tested [52], [53]. Data of relative abundance were also fitted to the power function [54], using nonlinear regression (Gauss-Newton method) (SAS version 8.02, SAS Institute Inc., Cary, USA, 2001). In this, *C_r_* = *a r^m^*; where *C_r_* is the relative abundance at rank *r* (a measure of taxon abundance relative to the abundance of other taxa), *r* is the abundance rank (the most abundant rank is given rank 1, the second most abundant one is 2, etc.), *a* is an empirical taxon- and location-specific constant and *m* is the shape parameter [55]. The standard error of the *m* parameter estimate *b_m_* was computed using the equation
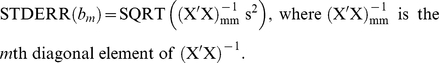



Under the hypothesis that *β_m_* is 0, the ratio *t* = [(*b_m_*)/(STDERR(*b_m_*))] is distributed as Student's *t* with degrees of freedom equal to the degrees of freedom for the mean square error.

The significance and fit were assessed by the F value of the non-linear regression and the non-linear coefficient of determination (pseudo-R^2^) for each rank/abundance curve, respectively. Differences in the relative abundance among stages, as well as among tuber starch content were calculated by two-sided t-tests.

Double dendrograms were generated using comparative functions and multivariate hierarchical clustering methods in NCSS 2007 (NCSS, Kaysville, Utah). The most abundant bacterial phyla and orders are included in the double dendrogram with clustering based on Ward's minimum variance and utilizing Manhattan distance calculations with no scaling. Here, the dendrogram linkages of the bacterial phyla and orders are not phylogenetic, but clustered based on the relative abundance among rhizosphere and bulk soil samples. Therefore, those samples with more similarity (less distance) are more closely related in overall bacterial diversity. Similarly, those bacteria that have similar percentages across all samples are more closely clustered.

## Supporting Information

Figure S1 **Ordination biplot diagrams generated by principal components analysis (PCA) of the bacterial communities in the rhizosphere.** Young plant - triangle, Flowering - filled square, Senescence - diamond. The eigenvalues displayed on the diagram axes refer to the percentage variation of ribotypes; environment correlation accounted for by the respective axis.
(TIF)

Figure S2 **Relative abundance of (A)** ***Alphaproteobacteria*** **(B)** ***Pseudomonas*** **(C)** ***Betaproteobacteria*** **(D)** ***Acidobacteria*** **in the rhizosphere of different potato cultivars and corresponding bulk soil, at different growth stages.** [A] Aveka, [Av] Aventra, [K] Karnico, [M] Modena, [P] Premiere, [D] Désierée, [bulk] bulk. Black bars – young plant, dashed bars – flowering, white bars – senescence.
(TIF)

Figure S3 **Double dendrogram, based on the Ward minimum variance clustering method for the ten most abundant genera investigated using 16S rRNA gene pyrosequencing.** The heat map indicates the relative percentage of each genus within each sample. On top of the figure, the distance of the samples based on weighted pair linkage and Manhattan distance methods with no scaling is shown, along with a distance score. [A] Aveka, [Av] Aventra, [K] Karnico, [M] Modena, [P] Premiere, [D] Désierée, [b] bulk, [yp] young plant, [f] flowering, [sn] senescence.
(TIF)

Table S1 **Primers with tags and adapters used in pyrosequencing.**
(DOC)

Table S2 **Evaluation of the fit of different distributions to pyrosequencing data from the rhizosphere and bulk soil community.**
(DOC)

Table S3 **The ten most abundant genera found in rhizosphere and bulk soil sample (- indicates no existance).**
(DOC)
