# Supplementary figures and images for: Comparative Analysis of Bacterial Communities in a Potato Field as Determined by Pyrosequencing
Source: PLoS One. 2011 Aug 19;6(8):e23321. doi: 10.1371/journal.pone.0023321 (PMC3158761; doi:10.1371/journal.pone.0023321)

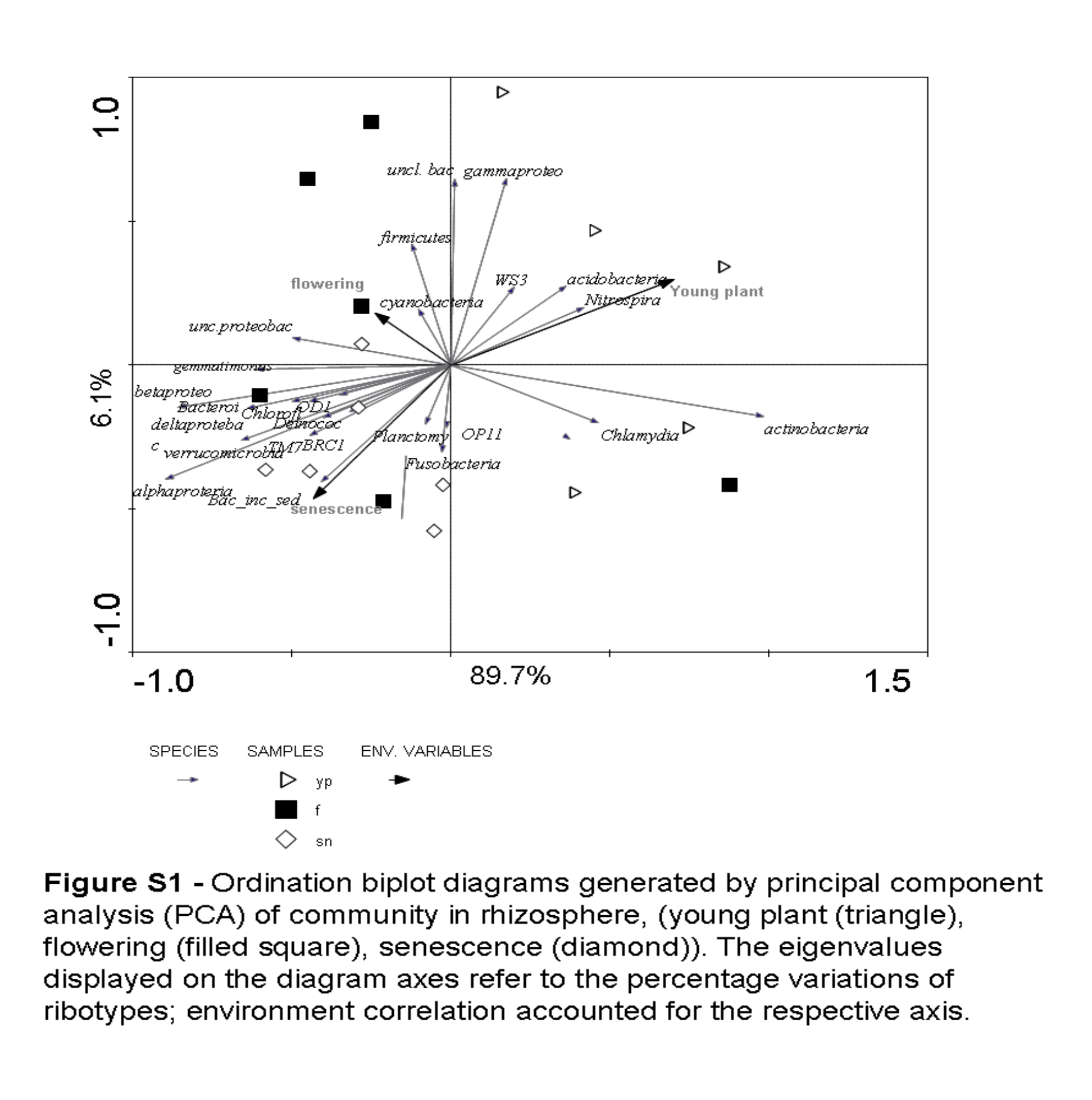

Supplement: Figure S1 — Ordination biplot diagrams generated by principal components analysis (PCA) of the bacterial communities in the rhizosphere. Young plant - triangle, Flowering - filled square, Senescence - diamond. The eigenvalues displayed on the diagram axes refer to the percentage variation of ribotypes; environment correlation accounted for by the respective axis. (TIF) [file pone.0023321.s001.tif]

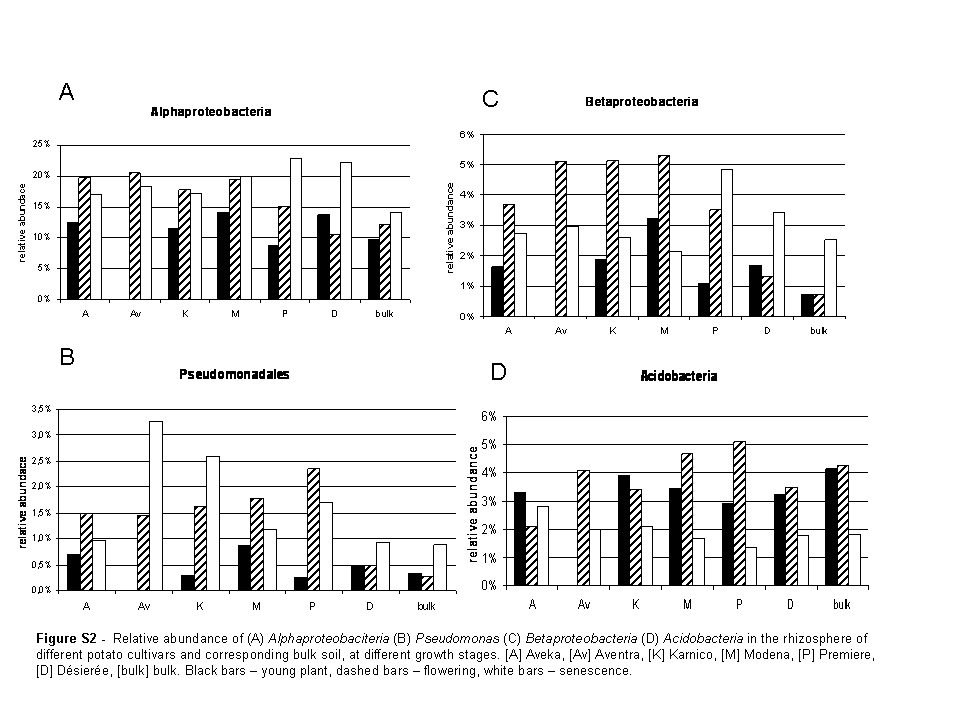

Supplement: Figure S2 — Relative abundance of (A) Alphaproteobacteria (B) Pseudomonas (C) Betaproteobacteria (D) Acidobacteria in the rhizosphere of different potato cultivars and corresponding bulk soil, at different growth stages. [A] Aveka, [Av] Aventra, [K] Karnico, [M] Modena, [P] Premiere, [D] Désierée, [bulk] bulk. Black bars – young plant, dashed bars – flowering, white bars – senescence. (TIF) [file pone.0023321.s002.tif]

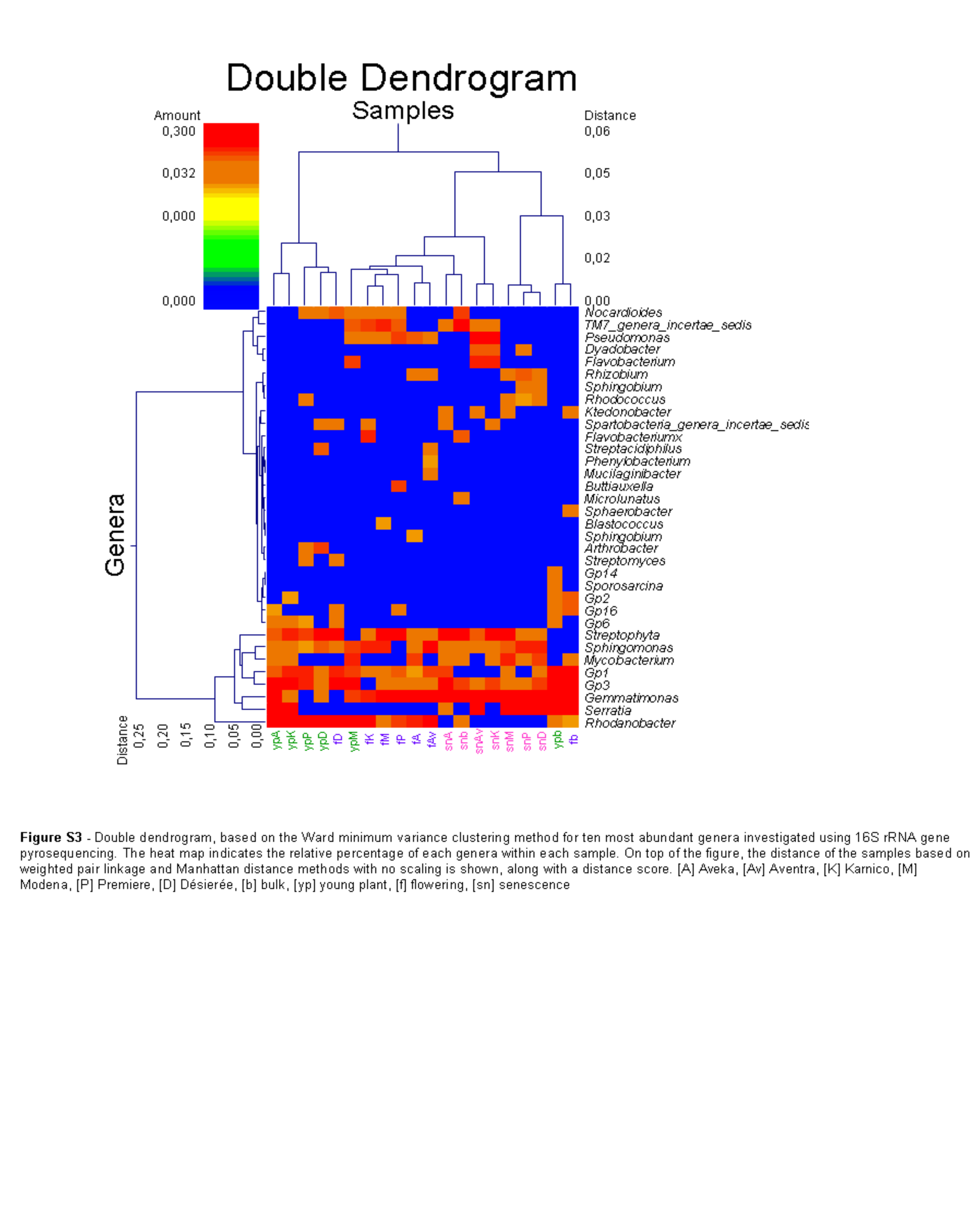

Supplement: Figure S3 — Double dendrogram, based on the Ward minimum variance clustering method for the ten most abundant genera investigated using 16S rRNA gene pyrosequencing. The heat map indicates the relative percentage of each genus within each sample. On top of the figure, the distance of the samples based on weighted pair linkage and Manhattan distance methods with no scaling is shown, along with a distance score. [A] Aveka, [Av] Aventra, [K] Karnico, [M] Modena, [P] Premiere, [D] Désierée, [b] bulk, [yp] young plant, [f] flowering, [sn] senescence. (TIF) [file pone.0023321.s003.tif]
