# Supplementary material for: Comparative Analysis of Bacterial Communities in a Potato Field as Determined by Pyrosequencing
Source: PLoS One. 2011 Aug 19;6(8):e23321. doi: 10.1371/journal.pone.0023321 (PMC3158761; doi:10.1371/journal.pone.0023321)
Supplement: Table S1 — Primers with tags and adapters used in pyrosequencing. (DOC) [file pone.0023321.s004.doc]

| Table S1. Primers with tags and adapters used in pyrosequencing. |
| --- |
| >**LinA_341F_1**  GCCTCCCTCGCGCCATCAG-ACGAGTGCGT-CCTAYGGGRBGCASCAG |
| >**LinA_341F_2**   GCCTCCCTCGCGCCATCAG-ACGCTCGACA-CCTAYGGGRBGCASCAG |
| >**LinA_341F_3**  GCCTCCCTCGCGCCATCAG-AGACGCACTC-CCTAYGGGRBGCASCAG |
| >**LinA_341F_4** GCCTCCCTCGCGCCATCAG-AGCACTGTAG-CCTAYGGGRBGCASCAG |
| >**LinA_341F_5** GCCTCCCTCGCGCCATCAG-ATCAGACACG-CCTAYGGGRBGCASCAG |
| >**LinA_341F_6** GCCTCCCTCGCGCCATCAG-ATATCGCGAG-CCTAYGGGRBGCASCAG |
| >**LinA_341F_7** GCCTCCCTCGCGCCATCAG-CGTGTCTCTA-CCTAYGGGRBGCASCAG |
| >**LinA_341F_8**  GCCTCCCTCGCGCCATCAG-CTCGCGTGTC-CCTAYGGGRBGCASCAG |
| >**LinA_341F_9** GCCTCCCTCGCGCCATCAG-TAGTATCAGC-CCTAYGGGRBGCASCAG |
| >**LinA_341F_10**GCCTCCCTCGCGCCATCAG-TCTCTATGCG-CCTAYGGGRBGCASCAG |
| >**LinA_341F_11** GCCTCCCTCGCGCCATCAG-TGATACGTCT-CCTAYGGGRBGCASCAG |
| >**LinA_341F_13**GCCTCCCTCGCGCCATCAG-CATAGTAGTG-CCTAYGGGRBGCASCAG |
| >**LinA_341F_14** GCCTCCCTCGCGCCATCAG-CGAGAGATAC-CCTAYGGGRBGCASCAG |
| >**LinA_341F_15** GCCTCCCTCGCGCCATCAG-ATACGACGTA-CCTAYGGGRBGCASCAG |
| >**LinA_341F_16** GCCTCCCTCGCGCCATCAG-TCACGTACTA-CCTAYGGGRBGCASCAG |
| >**LinA_341F_17** GCCTCCCTCGCGCCATCAG-CGTCTAGTAC-CCTAYGGGRBGCASCAG |
| >**LinA_341F_18** GCCTCCCTCGCGCCATCAG-TCTACGTAGC-CCTAYGGGRBGCASCAG |
| >**LinA_341F_19** GCCTCCCTCGCGCCATCAG-TGTACTACTC-CCTAYGGGRBGCASCAG |
| >**LinA_341F_20** GCCTCCCTCGCGCCATCAG-ACGACTACAG-CCTAYGGGRBGCASCAG |
| >**LinA_341F_21** GCCTCCCTCGCGCCATCAG-CGTAGACTAG-CCTAYGGGRBGCASCAG |
| >**LinB_806R**    GCCTTGCCAGCCCGCTCAG-GGACTACNNGGGTATCTAAT |
