# Supplementary material for: Comparative Analysis of Bacterial Communities in a Potato Field as Determined by Pyrosequencing
Source: PLoS One. 2011 Aug 19;6(8):e23321. doi: 10.1371/journal.pone.0023321 (PMC3158761; doi:10.1371/journal.pone.0023321)
Supplement: Table S2 — Evaluation of the fit of different distributions to pyrosequencing data from the rhizosphere and bulk soil community. (DOC) [file pone.0023321.s005.doc]

Table S2 Evaluation of the fit of different distributions to pyrosequencing data from the rhizosphere and bulk soil community

|  | [***χ***](http://en.wikipedia.org/wiki/Chi_(letter))**²** | | | **p-value*** | | |
| --- | --- | --- | --- | --- | --- | --- |
|  | **log series** | **truncated log** | **power law** | **log series** | **truncated log** | **power law** |
| **ypA** | 9,66 | 2,93 | 2,9 | 0,15 | 0,9 | 0,7 |
| **ypK** | 63,32 | 13,99 | 2,37 | **<0,001** | 0,1 | 0,5 |
| **ypM** | 39,17 | 39,309 | 7,83 | **<0,001** | **<0,001** | 0,2 |
| **ypP** | 10,92 | 6,96 | 1,94 | 0,1 | 0,5 | 0,75 |
| **ypD** | 12,25 | 7,45 | 8,1 | **>0,05** | 0,5 | 0,15 |
| **fA** | 5,33 | 10,25 | 10,02 | >0,5 | 0,3 | 0,1 |
| **fAv** | 1,66 | 21,83 | 8,6 | 0,98 | **0,01** | 0,2 |
| **fK** | 4,42 | 9,5 | 10,48 | 0,8 | 0,2 | 0,06 |
| **fM** | 6 | 16,71 | 10,3 | 0,5 | **<0,05** | 0,06 |
| **fP** | 15,6 | 19,72 | 10,7 | **<0,05** | **0,02** | 0,06 |
| **fD** | 6,9 | 4,72 | 1,83 | >0,3 | 0,7 | 0,8 |
| **snA** | 3,82 | 8,6 | 2,81 | 0,8 | 0,4 | 0,8 |
| **snAv** | 4,06 | 1,71 | 5,39 | 0,7> | 0,99 | 0,4 |
| **snK** | 3,73 | 5,49 | 2,19 | 0,8 | 0,7 | 0,7 |
| **snM** | 4,73 | 10,84 | 2,68 | 0,7 | 0,2 | 0,7 |
| **snP** | 14,16 | 36,67 | 7,18 | **<0,05** | **0,001** | 0,2 |
| **snD** | 4,9 | 3,7 | 1,85 | 0,7 | 0,9 | 0,75 |
| **bs** | 10,85 | 6,35 | 5,41 | 0,2 | 0,6 | 0,06 |
| **bf** | 8,38 | 8,66 | 1,26 | 0,2 | 0,3 | 0,85 |
| **bsn** | 10,58 | 165 | 4,89 | 0,2 | **0,001** | 0,5 |
| *****Values above 0.05 indicate a failure to reject null hypothesis that two distributions are the same.  [A] Aveka, [Av] Aventra, [K] Karnico, [M] Modena, [P] Premiere, [D] Désierée, [b] bulk, [yp] young plant, [f] flowering, [sn] senescence | | | | | | |
