# Supplementary material for: Comparative Analysis of Bacterial Communities in a Potato Field as Determined by Pyrosequencing
Source: PLoS One. 2011 Aug 19;6(8):e23321. doi: 10.1371/journal.pone.0023321 (PMC3158761; doi:10.1371/journal.pone.0023321)
Supplement: Table S3 — The ten most abundant genera found in rhizosphere and bulk soil sample (- indicates no existance). (DOC) [file pone.0023321.s006.doc]

Table S3 The ten most abundant genera found in rhizosphere and bulk soil sample (- indicates no existance)
